# Extraction, characterization and properties evaluation of pineapple leaf fibers from Azores pineapple

**DOI:** 10.1016/j.heliyon.2024.e26698

**Published:** 2024-02-20

**Authors:** Diego M. Chaves, Joana C. Araújo, Carina V. Gomes, Sónia P. Gonçalves, Raul Fangueiro, Diana P. Ferreira

**Affiliations:** a Centre for Textile Science and Technology (2C2T), University of Minho, 4800, Guimarães, Portugal; b CEB - Centre of Biological Engineering, University of Minho, 4710-057, Braga, Portugal; c Department of Textile Engineering, University of Minho, Guimarães, Portugal

**Keywords:** Pineapple leaf fibers, Azores pineapple wastes, Biological retting, PALF mechanical properties, Long fibers

## Abstract

Pineapple leaves can provide competitive and high-quality fibers for textile purposes. Despite pineapple being cultivated in the Portugues islands there is still a technology gap for the extraction and treatment of Pineapple Leaf Fibers (PALF) in Europe. Since Azorean Pineapple differs significantly from other plants in the bromeliad family, the properties and characterization of its leaf fibers were explored for the first time. Long fibers have been extracted by hand scraping and compared to biological retting at 25 °C for different time periods. It was explored the properties of PALF from plants of different ages (11- and 18-months) and from different zones of the leaves (beginning, middle, and tip). Physical-mechanical properties of Azores PALF were determined, including diameter, linear density, strength, Young's modulus, and elongation at break and characterized by ATR-FTIR, XRD, TGA/DTG, and FESEM to understand their chemical and morphological characteristics. While slight differences were observed between different ages, variations in physical-mechanical properties were notable among fibers extracted from different leaf positions. Extraction of Azores PALF through 25 °C biological retting for 14 days effectively eliminated non-fibrous matter and produced the thinnest and strongest fibers. These fibers ranged between 34.9 and 168.3 μm in diameter, 1.39 and 7.07 tex in linear mass density, 37–993 MPa in tensile strength, 1.0–3.9 % in elongation at break, and 2.4–21.8 GPa in Young's modulus.

## 1 Introduction

Circular economy principles have gained significant attention in scientific studies, especially in the textile industry's agro-industrial waste valorisation. This approach aims to address the pressing concerns of human dependence on fossil materials and its adverse environmental impacts. By embracing circular economy practices, the textile industry seeks to reduce waste generation, minimize resource consumption, and create sustainable solutions for waste management [1].

Natural fibers, primarily composed of cellulose, offer a promising alternative to conventional synthetic fibers [2]. These eco-friendly fibers have the potential to partially or completely replace their synthetic counterparts, contributing to a more sustainable textile industry. Beyond the well-known sources such as cotton, natural fibers can be extracted from various waste materials like hemp stems [3,4], banana trees [[5], [6], [7]] and pineapple leaves [8,9].

Pineapple has become one of the most produced tropical fruits across the globe. According to data from the Food and Agriculture Organization (FAO) global pineapple production reached 28,6 million tons in 2021 [10]. Countries like Costa Rica (2,9 million tons), the Philippines (2,9 million tons), Brazil (2,3 million tons), Thailand (1,8 million tons) and India (1,8 million tons) led the global pineapple production in 2021. However, with such extensive cultivation, there inevitably comes a significant amount of waste posing both environmental and economic challenges [11]. Pineapple leaves are typically managed as a waste and left on the ground or even burned after pineapple harvest. However, these wastes can improve circular economy since they can provide high-quality long fibers (Pineapple Leaf Fibers – PALF) for textile purposes and avoid food competition [12]. Moreover, its utilization supports local communities and adds value to agriculture.

PALF can be processed to be used in the production of a wide range of products: cellulose nanofibers for heavy metal ions removing and prosthetic heart valves [[13], [14], [15]], tile composites [16], yarns [17] reinforced rubber [18], sound absorber [19], aerogels for heat and sound insulations [20], soil reinforcement [21] and handcrafts. PALF presents a low cost (360–550 US$/ton) [22], low density (0.80–1.60 g/cm^3^) [23], high tensile strength (126,6–1627,0 MPa) [24], and can easily retain dyes [25], if compared to others natural fibers. Additionally, theyare renewable and biodegradable, offering a sustainable and eco-friendly alternative to traditional textile materials. Due to its unique properties, PALF is gaining increasing importance in textile applications and can be used alone or blended in the form of yarns or non-woven fabrics, for the production of apparel [26], handbags, accessories and footwear [27], soil covering [9], thermal and sound absorbers [28,29], and fabric-reinforced composites [30].

PALF can be extracted as short or long fibers. The extraction of long fibers offers a several number of advantages over short fibers extraction. Long fibers are suitable for making composites [24], unidirectional structures [31,32] and handicrafts. Long fibers also cover short fiber applications since it is easy to chop them to shorter lengths when needed. These fibers also make possible ring spinning, which generally produces high-quality yarn with better strength, evenness, and smoothness, over than other spinning methods [12].

PALF properties can vary with the plant age, variety, extraction method or position in the leaf [9,33,34]. Therefore, these characteristics are very important to not only evaluate the most suitable extraction methods, but also to better understand their potential of applications. While pineapples are cultivated on Portuguese islands (*i.e*., Azores islands) and European companies have sell products made from PALF [27], there is still a technological gap in fiber extraction and treatments, culminating in the absence of a PALF supply chain. As a result, the raw material continues to be imported from Asian countries, affecting its environmental footprint.

The annual production of pineapple in Portugal is around 1 million tons per year [10], generating thousands of wastes each year. Despite this, the authors were not been able to find any published information regarding the extraction as well as the physical-mechanical properties of PALF from Azores as of this date, which demonstrates the novelty of this work. Therefore, in this work, the physical and mechanical properties of PALF extracted from wastes from pineapple cultivation in the Azores Islands were studied. Some important parameters were explored, as different plant ages (*i.e.,* 11- and 18-months), different positions in the leaf and the conventional scraping method was compared with biological retting at 25 °C. The effects of these parameters on the surface and morphology of fibers were assessed using the Field Emission Scanning Electron Microscopy technique (FESEM). Changes in the physical and chemical lignocellulosic structure of the raw fibers were investigated through X-ray Diffraction (XRD), Attenuated Total Reflectance Fourier Transformed Infra-Red Spectroscopy (ATR-FTIR), Thermal Gravimetric (TGA), and Differential Thermal Gravimetric analysis (DTG). The extracted PALF using the best conditions were characterized regarding their mechanical properties, i.e., tensile strength, elongation at break, and Young's Modulus, determined.

## 2 Experimental

### 2.1 Materials

Fresh and green pineapple leaves, harvested from plants of Cayene variety with 11- and 18-months age, were supplied by Boa Fruta Company, from São Miguel Island, Azores, Portugal.

### 2.2 Extraction of Azores PALF

In order to understand the effect of the extraction method, the extraction of PALF was carried out by two methods: hand scraping and retting at 25 °C. Hand scraping consists of the removal of the non-fibrous matter by scraping the leaf surface until fibers are exposed. The back of a knife against a wood board was used to hand scrape 11- and 18-months age leaves [35]. Biological retting involves bacterial decomposition of the non-fibrous matter, which acts as a glue among the fibers. A bath of fresh 11-months aged leaves was soaked in pure water, kept at 25 °C for 28 days [36], and samples were taken each 7 days and scraped to obtain the fibers. Degummed samples were extracted from 8 randomly collected leaves, and the material-to-liquor ratio was maintained at 1:8 until the end of the experiment. The fresh bundle was washed in water to remove remaining impurities and then dried outdoor for 5 days. The dried bundle was then manually combed to open the fibers.

### 2.3 Characterization of Azores PALF

Characterization of PALF was carried out to access morphology and structural changes of the extracted fibers using different techniques.

#### 2.3.1 Optical microscopy (OM)

Micrographs of the extracted fibers as well as of the fermentation liquor from the retting procedure were taken in a Leyca Microsystems Binocular DM750 M Microscopy (Wetzlar, Germany) with a high definition digital camara attached. The diameters of the fibers were measured from the micrographs through the software ImageJ.

#### 2.3.2 Attenuated total reflectance-Fourier transform infrared spectroscopy (ATR-FTIR)

ATR-FTIR analysis was employed to monitor chemical changes occurring in the lignocellulosic structure of the fibers. Analysis was performed in a Shimadzu Fourier Transform Infrared Spectrophotometer IRAffinity-1S (Kyoto, Japan). Spectra were collected over 45 scans and in the spectral range 4000-400 cm^−1^, with a resolution of 4 cm^−1^ in transmittance mode.

#### 2.3.3 Field Emission Scanning Electron Microscopy (FESEM)

Surface and morphologic analysis of the extracted PALF was carried out by FESEM in a FEI NOVA 200 Nano SEM (Hillsboro, OR, USA). To make the surface conductive, the samples were coated with a film of Au and Pd (80:20), before the analysis, in a Cressington 208HRD High Resolution Sputter Coater. FESEM images were taken using secondary electron mode with an accelerated voltage of 10 kV and Everhardt Thornley Detector (ETD).

#### 2.3.4 Thermogravimetric analysis and differential thermogravimetric analysis (TGA/DTG)

Thermogravimetric analysis was used to access changings in the content of holocellulose fraction. TGA/DTG of the fibers were performed in a Hitachi STA7200RV Thermal Analysis System (Chiyoda, Tokyo, Japan), in the range of 30–600 °C at 10 °C min^−1^. N_2_ flux was 200 mL min^−1^ and initial sample weighted between 5.1 and 6.6 mg. Moisture content of the extracted PALF was estimated by proximate analysis of the first weight loss where a constant weight was reached (*i.e.*, 120 °C) [37].

#### 2.3.5 X-ray diffraction (XRD)

In order to determine the crystallinity index of the cellulose fraction, the diffraction pattern was collected with a Bruker AXS D8 Discovery XRD instrument, (Karlsruhe, Germany). Diffractometer operated using Cu-Kα as radiation source (wavelength of 1.5406 Å), accelerating voltage at 40 kV, current at 40 mA, and 2 θ angle ranging from 5 to 60° at 1° min^−1^. Cristality index (I_c_) was calculated according the peak height method reported by Segal et al. [38], through the following equation (1):

(1) $$I_{c}=\frac{\left(I_{002}-I_{am}\right)}{I_{002}}\;x\;100$$

where I_002_ is the maximum intensity of the crystalline peak associated to the plane (002), at 2 θ = 22.3°, and I_am_ is the minimum intensity found for the peaks (002) and (101), at 2 θ = 18.5°.

### 2.4 Evaluation of physical-mechanical properties of Azores PALF

Fiber bundles were kept at 20 ± 1 °C and 65 ± 1 % of atmosphere moisture for 24 h in a climatic chamber Aralab Fitoclima 150 EDTU (Rio de Mouro, Portugal), before procedures according to standard ASTM D3822/D3822M − 2014. Linear density tests of the fibers were performed before tensile tests and adapted from ISO 07211-5-1984, 2002 [40]. Individual fibers were stretched at 5 gf in a Sodemat Crimp Tester, for length measuring, and then weighed. The linear density was given by the ratio between weight (g) and length (km). Tensile tests were performed for 35 individual fibers in a universal Hounsfield CRE Testing Machine H100KS (Redhill, England) following standard parameters for fibers [39], using a load cell of 250 N and maintaining 50 mm test length and 1 mm min^−1^ test speed. Tensile properties (*i.e*., tensile strength, Young's modulus and elongation at break) of the fibers were measured.

## 3 Results and discussion

### 3.1 Azores pineapple plant and extraction of PALF

**Fig. 1:**
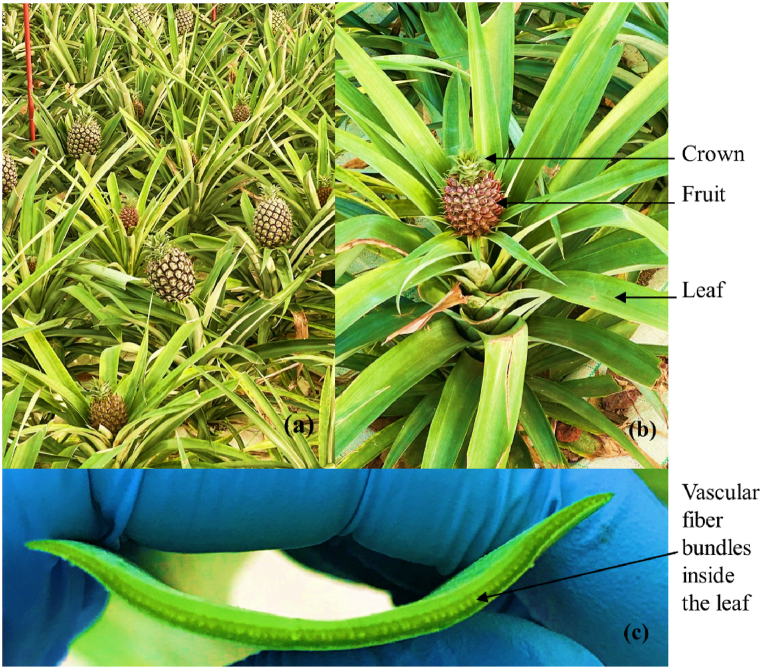
Azores pineapple plants: (a) Plants grew up in greenhouse, (b) parts of the plant and (c) transverse-section leaf with exposed vascular fiber bundles.

The Azores pineapple is a unique and valued variety of pineapple grown in the Azores archipelago, an autonomous region of Portugal in the North Atlantic Ocean that presents a favourable climate for agriculture (Fig. 1).

The pineapple is primarily grown in greenhouses to protect the delicate fruit from the unpredictable weather conditions in the Azores. In terms of appearance, the Azores pineapple is smaller than some other commercially grown pineapples [41], with a more concentrated flavour. The outer skin is golden-yellow with a rough, hexagonal pattern, and the crown of the fruit has a vibrant green colour (Fig. 1-b). The PALF is abundantly present in the pineapple leaf, arranged in parallel bundles that run along the leaf's length and are uniformly distributed across its width, as clearly depicted in Fig. 1-c. According with Surajarusarn et al. (2019) two distinct regions within the leaf where fiber bundles were prominent can be distinguished. Firstly, in the vascular tissue located at the center of the leaf, and secondly, in the mesophyll tissue adjacent to the lower surface. This insightful observation sheds light on the specific localization of PALF within the leaf's structure.

**Fig. 2:**
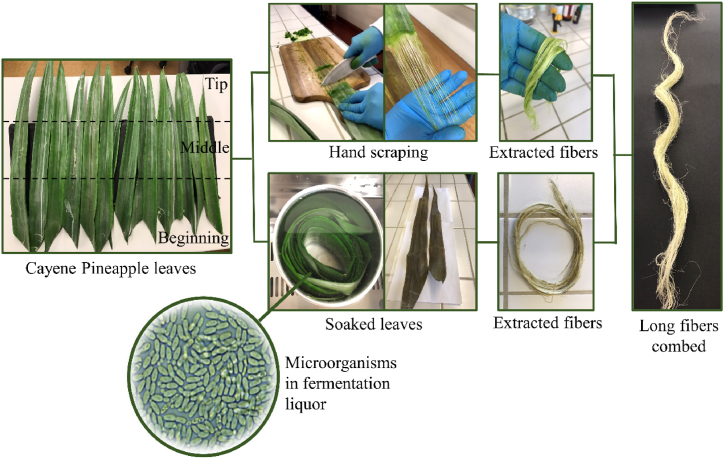
Schematic diagram of the different zones of the leaves and experimental procedures to extract long PALF.

The conditions for hand extraction and retting protocols, such as time and temperature, can vary depending on the plant variety [42,43]. Since the extraction of PALF from the Azores has not been reported yet, it is necessary to study the optimization as well as the effect of these methods on the quality of the extracted fibers. The experimental procedures of long PALF extraction are summarized in a scheme of Fig. 2.

It was possible to extract long fibers from any position of the leaves, i.e., beginning, middle and tip (Fig. 2). Making comparisons among the properties of natural fibers extracted from different zones of the same leaf is essential for understanding the variability within a plant species, optimizing resource use, and tailoring fiber selection to specific applications [34]. The pineapple leaves used in this work weighted between 21.9 g and 53.1 g and measure between 5.7 cm and 7.4 cm in width, 55.5 cm and 96.5 cm in length and up to 2.4 mm in thickness.

The retting process has changed the aspect of the leaves possibly as a consequence of the microbial degradation of non-fibrous matter. The leaves became brown, and the liquor presented a characteristic smell of the fermentation process with a high concentration of Bacillus bacteria (Fig. 2). These bacteria are usually present in the complex microbial community, which is usually responsible for the removal of pectin, hemicellulose, and other non-fibrous matter from natural fibers during the retting process, and they have been isolated and identified by some researchers [44].

As we can see in Fig. 2, both extraction methods provided long fibers (*i.e*., up to 70.8 cm long). Regardless of the age of the plant and the extraction method, the speed of manually extraction and productivity (i.e., average amount of extracted fiber per leaf) of fibers were close to 10 min/leaf and 0.3 g/leaf, respectively (Table 1). However, it is worth noting that smaller values are observed for PALF extracted from leaves retted for 28 days. This outcome strongly suggests that prolonged retting period of 28 days has resulted in damage to the cellulosic fibers. Consequently, during the subsequent manual scraping process, these weakened fibers are more prone to breakage and fragmentation, leading to reduced productivity of long PALF.

**Table 1:** Data of the extraction procedures.

Age of the plant (months)	Extraction method/Retting time (days)	Speed of scraping (min/leaf)	Productivity (g/leaf)	Yield (% w/w)
11	Hand scraping	10	0.2653	0.8
18	Hand scraping	10	0.3288	0.8
11	Retting at 25 °C (7)	8.5	0.2916	0.8
	Retting at 25 °C (14)	9.0	0.3482	0.9
	Retting at 25 °C (21)	11	0.2814	0.9
	Retting at 25 °C (28)	5.6	0.2079	0.8

The PALF yield, representing the weight of PALF per weight of fresh leaves, ranges from 0.8% to 0.9% w/w. Although there are no adequate values for comparison in the literature, as these data are not published for PALF of the Azores, it is notably that pineapple leaves contain a substantial amount of water and other non-fibrous matter. This uniformity in results, despite the age of the plant or extraction method, can be attributed to the manual scraping method, which was used even after the retting procedure. Accurately measuring the force during manual scraping is challenging and may inadvertently rupture fibers that end up retained in the extracted pulp, ultimately reducing the actual yield—this observation has been duly noted. It's essential to recognize, however, that the manual scraping method was employed primarily to provide PALF on a laboratory scale. The objective of this work was to study Azores' PALF properties and the fiber yield will be optimized in future work with the mechanization of the process.

### 3.2 Characterization of Azores PALF

Long fibers were extracted from leaves of plants with age of 11 and 18 - months, using different extraction methods (hand scraping and water retting). It was also possible to make comparisons between fibers extracted from different zones in the leaves (see Fig. 2). The full characterization of the different PALF obtained in this work, as well as the comparative study between them, is presented next.

#### 3.2.1 ATR-FTIR analysis

**Fig. 3:**
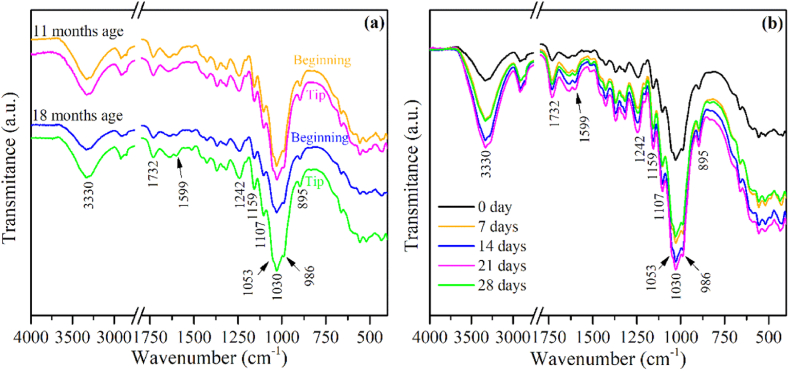
ATR-FTIR analysis of PALF: **a)** ATR-FTIR spectra from different regions of the 11 and 18-months aged PALFs extracted by hand scraping; **b)** ATR-FTIR spectra of the PALFs extracted by retting at 25 °C for 7, 14, 21 and 28 days.

The ATR-FTIR spectra of the extracted fibers are shown in Fig. 3, No significant distinctions were observed in the ATR-FTIR spectra of fibers aged 11 and 18 months, as well as across different regions (Fig. 3). These spectra exhibit the distinctive absorption bands associated with cellulose, hemicellulose, and lignin fractions, indicating their presence within the fibers. The absorption bands present in the spectra were assigned as follows: 3330 cm^−1^ (-OH, cellulose and hemicellulose) [25,45], 1732 cm^−1^ (C=O, hemicellulose acetyl and uranic ester groups) [18,46], 1599 cm^−1^ (C=C, aromatic skeleton of lignin) [25,47], 1242 cm^−1^ (-COO, hemicellulose) [48], 1159, 1107 cm^−1^ (C–*O*–C, β-1,4 glycosidic linkage of cellulose) [36,48], 1053 cm^−1^ (C–O, C-6 of pyranose ring in cellulose) [48], 1030 cm^−1^ (C–O and C–*O*–C, pyranose ring in cellulose) [18,45], 986 and 895 cm^−1^ (C–O and C–H, cellulose) [18,46].

In the ATR-FTIR analysis of PALF extracted from retted leaves at 25 °C, it is evident that the bands associated with cellulose, hemicellulose, and lignin remain consistent regardless retting time. However, an interesting observation is that the intensity of these bands progressively increases until reaching a peak at 21 days of retting, as depicted in Fig. 3. While all the absorption bands of the fiber's components for 21 days exhibit a higher intensity, they closely resemble the absorption bands for 14 days. This implies that the removal of non-fibrous components from the fiber bundles, facilitated by microbial degradation, becomes more effective as time progresses, reaching an optimal state at the 14 days of retting.

A notable decrease is observed from 21 days to 28 days band's intensity, overlapping the 7-days bands (Fig. 3). This decrease in intensity suggests potential damage to the fibers extracted from leaves that underwent a 28-day retting process, further corroborating the findings presented in Table 1.

#### 3.2.2 TGA/DTG analysis

**Fig. 4:**
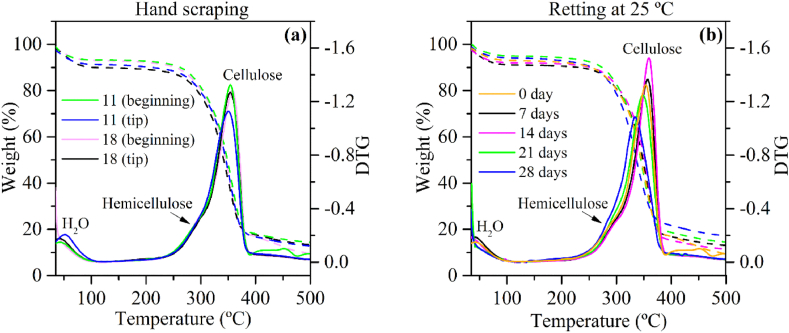
Thermogravimetric analysis of PALF extracted by different methods: **a)** TG-DTG curves from different regions of the PALF from hand-scraped leaves with 11- and 18-months age; **b)** TG-DTG curves of the PALF from retted leaves at 25 °C for 7, 14, 21 and 28 days.

The TGA/DTG curves of the extracted PALF exhibit a similar shape to those previously reported in the literature for PALF [49]. TGA/DTG curves are presented in Fig. 4.

Notably, three distinct thermal events can be observed. The first DTG peak, ranging from 30 to 120 °C, corresponds to the release of moisture. The main DTG peak, spanning from 200 °C to 380 °C, predominantly represents the thermal decomposition of cellulose. Additionally, the thermal decomposition of hemicellulose can be observed as a shoulder between 200 °C and 300 °C.

The moisture control of PALF is essential for its processing as well as for its applications. Moisture control is vital in natural fibers reinforced composites materials to ensure proper adhesion and mechanical properties [50]. Numerous hydrogen bonds within the plant fiber's cell wall break upon exposure to atmospheric moisture, leading hydroxyl groups to form new bonds with water molecules. Consequently, the reinforcement of hydrophilic fiber with a hydrophobic resin fiber result in swelling within the matrix, causing weak fiber-matrix bonding, dimensional instability, matrix cracking, and diminished composite mechanical properties [51]. In the production of textiles using natural fibers, proper moisture control ensures the fibers are pliable and suitable for spinning and weaving. A high moisture content can lead to degradation of stored fiber quality due microbial growth, while excessively low moisture can result in brittle fibers and reduced fiber strength [52]. Insufficient moisture levels can lead to the generation of static electricity through friction and also result in the attraction of dust and dirt [52].

The moisture content of PALF fibers extracted by hand scraping was 7.2% at the beginning and 10.3% at the tips for 11-month-old plants. For 18-month-old plants, it was 6.8% at the beginning and 8.9% at the tips of leaves. Moisture content notably decreased in fibers extracted from older plants and from the initial region as compared to the tip. Razali et al. observed similar results in their study on the impact of fiber maturity on Roselle fibers and they attributed the finds to the lumen size and plant age [53]. Lumen sizes are related to the water absorption; as fiber age the cell wall increases in thickness and the lumen size decreases [54,55]. Therefore, older plants present a thicker cell wall, a smaller lumen and consequently a lower moisture content, as observed in this work.

For PALF fibers extracted by retting at 25 °C, the moisture content was 7.6%, 9.0%, 8.0%, 5.2%, and 6.1% for 0, 7, 14, 21, and 28 days, respectively. The higher moisture content observed in fibers extracted between 7 and 14 days can be attributed to increased holocellulose content. This observation aligns with the increase observed in (-OH) absorption bands in FTIR. Holocellulose absorbs water due to the presence of hydrophilic groups, such as hydroxyl (-OH) and carboxyl (-COOH) groups, forming hydrogen bonds with water molecules. This increase in holocellulose content is a result of the removal of non-fibrous material. Hence, the reduction in moisture content after 21 days may be due to the decomposition of this fraction, likely caused by microbial activity resulting from prolonged exposure of the fibers.

The moisture content in the extracted PALF is similar to the equilibrium moisture content reported in the literature (approximately 9 wt%) [56], indicating that the fibers can be transported, stored, and used with their current moisture content for various applications, such as PALF-based yarns [57] or PALF-based composites [58].

The DTG curves obtained from hand-scraped leaves of different ages (Fig. 4-a)**,** reveal a smaller cellulose DTG peak at the tip region of the fibers extracted from plants with 11 months of age. This observation suggests a reduced cellulose content at the tip of long PALF from younger pineapple plants, an intrinsic feature of natural fibers. The cells at the tip are still undergoing maturation and have lower cellulose synthesis [59].

Observing the DTG curves of PALF extracted from the retted leaves (Fig. 4-b), a noteworthy trend emerges. There is a distinct shift to higher temperatures and increase in the cellulose DTG peak from 0 to 14 days of retting. Similar results were observed by Mazian et al. while studying the effect of field retting time on the properties of hemp fibers [60]. It is stated that an increase in the retting duration leads to an increase in the decomposition temperature of the fibers. Removing non-cellulose components improves the structural order of cellulose, strengthening the intramolecular and molecular hydrogen bonds that require higher temperatures to be broken down. These findings also indicate an increase in cellulose content during this period. However, after 14 days of retting, the cellulose DTG peak undergoes a decrease and shifts to lower temperatures. Lignocellulosic materials, such as natural fibers, are renowned for their high recalcitrance, because of robust intermolecular and intramolecular bonds within and among the main fractions: cellulose, hemicellulose, and lignin [61]. A shift in the cellulose DTG peak towards lower temperatures signifies a loss in thermal stability due to the breakdown of recalcitrance [62] and a decrease in the intensity of the DTG peak related to cellulose reflects a reduction in cellulose content [60]. These insights from DTG analysis imply that periods of retting longer than 14 days (i.e., 21 and 28 days) represent prolonged exposure times for the leaves. During this extended duration, the actions of microorganisms contribute to the weakening of the lignocellulosic structure of the fibers, allowing them access to and enabling an attack on the cellulose fraction, resulting in damage to the PALF structure integrity.

The TG/DTG analysis aligns with the findings of the ATR-FTIR analysis, providing further support for our conclusions. These results strongly suggest that a retting period of 14 days enhances the removal of non-fibrous matter, resulting in increased cellulose content. On the other hand, a prolonged water retting period of 28 days appears to be excessive, leading to potential damage of the fibers.

#### 3.2.3 XRD analysis

**Fig. 5:**
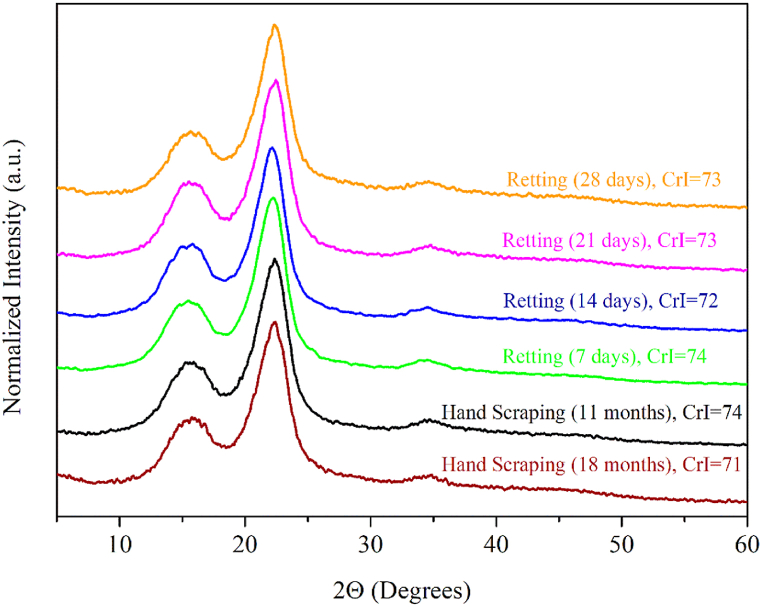
Diffractograms of 11 and 18-months aged fibers extracted by hand scraping and fibers extracted from retted leaves at 25 °C for 7, 14, 21 and 28 days.

XRD analysis were performed in order to investigate the proportion of crystalline cellulose. The diffractograms are shown in Fig. 5**,** as well the calculated crystallinity index (CrI).

Observing the diffractograms presented in Fig. 5, it is possble to notice that they exhibited simmilar diffraction peaks, which are characteristic of vegetal natural fibers, due to the presence of cellulose as the main component [63,64]. Cellulose can exist in either amorphous or crystalline form, varying in its level of crystallinity [65]. A higher degree of crystallinity in cellulose structure is associated with the improvement of mechanical strength, stiffness, and thermal stability, making it more resistant to biodegradability. The alignment of cellulose microfibrils contributes significantly to the strength and stiffness of natural fibers. When the microfibrils are aligned along the longitudinal axis of the fiber, it enhances the overall tensile strength and rigidity of the material [66]. Variations in the cellulose structure (i.e., crystallinity and microfibril alignment) influence the diffraction peaks. The observed uniformity among the diffraction peaks in various samples provides valuable insights and indicates that there were no changes in the original crystalline structure of cellulose whithin the fibers, regardless of plant age or extraction method. Consequently, the alterations observed in the mechanical properties of the fibers were not influenced by changes in the cellulose structure.

Generally, the crystallinity index of natural fibers can be enhanced through various treatments such as acid hydrolysis, mercerization or bleaching. Strong acids break down the amorphous regions of cellulose, leaving behind crystalline cellulose. By removing non-crystalline components, acid hydrolysis increases the proportion of crystalline cellulose [52,67]. Mercerization uses a solution of NaOH for the removal of the surface's impurities, disintegration of the middle lamella, and/or interfibrillar swelling. The removal of the interfibrillar region induces crystalline rearrangement, resulting in higher crystallinity [68]. Bleaching involves the use of chemicals like alkaline H_2_O_2_ or NaOCl to remove impurities, color, and residual non-cellulosic substances from natural fibers. By removing impurities and amorphous regions that can interfere with the regular packing of cellulose chains, bleaching enhances the crystallinity of fibers [69].

Crystallinity index values ranged between 71 and 74. It's possible to observe that there aren't any significant differences among them. The less pronounced effect of the fiber extraction method on the proportion of crystalline cellulose can be attributed to the lower exposure of fibers during the retting process compared to treatments like degumming. Extraction by retting provides a relatively protective environment for the fibers within the retting medium, while degumming involves more direct exposure to chemical agents or mechanical forces. Consequently, the retting process did not remove hemicellulose and lignin fractions, as indicated by ATR-FTIR analysis, preserving the proportion between non-crystalline and crystalline cellulose. This distinction in exposure during extraction methods explains the relatively minimal impact on the crystallinity index of the PALF fibers observed in this study.

### 3.3 Surface and morphology analysis of Azores PALF

**Fig. 6:**
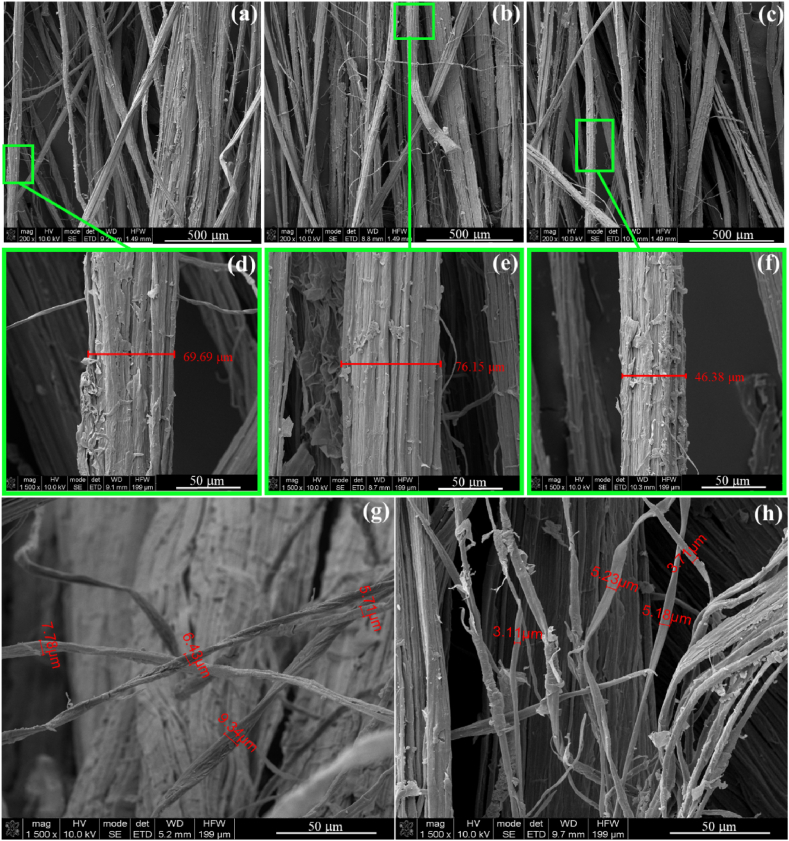
FEG-SEM images of PALF extracted by different methods and from plants of different ages: (a), (d) 11-months aged hand scraped fibers at 200× and 1,5000× magnification, (b), (e) 18-months aged hand scraped fibers at 200× and 1,5000× magnification, (c), (f) fibers from leaves retted at 25 °C for 28 days at 200× and 1,5000× magnification (g) and (h) elementary fibers from hand scraped 18 and 11-months aged leaves at 1500× magnification, respectively.

SEM micrographs were used for surface and morphological analysis of the extracted fibers. Surface images of fibers extracted from plants at different ages and by different methods are shown in Fig. 6.

In FESEM micrographs, captured at 200× magnification (Fig. 6-a, 6-c and 6-e), the pineapple leaf fibers exhibited a long and slender appearance, with diameters ranging from tens to hundreds of micrometers. Remarkably, the morphology of the extracted fibers remains constant, regardless of the extraction method or plant age. Zooming in at 1,500× magnification (Fig. 6-b, 6-d and 6-f), the surface texture becomes more evident, revealing a rugged and uneven structure consisting of closely packed fibrils. We also observe the presence of impurities on the surface, not removed during the extraction process, such as waxes, resins, and other natural substances. These impurities are common on surface of only extracted PALF [70] and further treatments are required to remove them and defibrillate PALF.

Interestingly, the micrographs also reveal differences in the fiber diameter. Specifically, the PALF extracted by hand scraping from leaves with 18-month age exhibits fibers with larger diameters (*i.e*., 76.15 μm), while fibers from the retting process for 28 days at 25 °C display smaller diameters (*i.e*., 46.38 μm). These observations provide valuable insights into the intriguing and complex nature of pineapple leaf fibers, underscoring their potential for diverse applications in textiles and composite materials.

A fascinating observation emerges as we delve deeper into the structure of pineapple leaf fibers. While the fibers initially appear long and individual, they reveal themselves to be bundles of elementary fibers, each possessing a flattened shape with diameters ranging from 3.11 to 5.23 μm (Fig. 6-h) for fibers from 11-months aged leaves and 5.71–9.34 μm (Fig. 6-g) for 18-months aged.

**Fig. 7:**
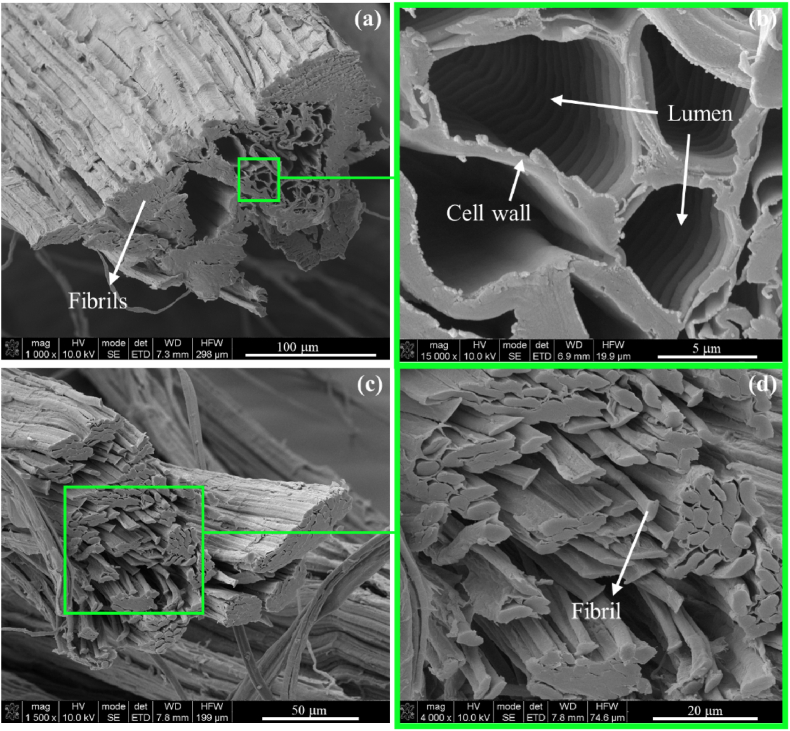
PALF cross sectional FEG/SEM images of 11-months aged hand scraped fibers at (a) 1000 × and (b) 15,000× magnification and fibrils bundle at (c) 1500× and (d) 4000× magnification.

Cross-sectional FEG-SEM micrographs were also collected for extracted PALF (Fig. 7).

As observed in the cross-section of an individual fiber at 1000× magnification (Fig. 7-a), PALF exhibits a distinctive hollow interior running along its length. Zooming in at 15,000× allows us to clearly discern the lumen and the cell wall (Fig. 7-b), which together form the cavities within PALF, contributing to its lightweight (*i.e*., 0.732–1.526 g cm^−3^) [71,72] and excellent sound and thermal insulation properties [19,28]. Similar observations for the cross sections of PALF were already related in literature for PALF extracted from Colombian pineapple [73]. Moreover, the micrographs reveal that the hollow center of the fiber is surrounded by bundles of elementary cellulosic fibers, known as fibrils (Fig. 6-c). These fibrils are bound together by cementitious materials such as hemicellulose and lignin (Fig. 6-d) [70]. Understanding this finer level of morphology can significantly impact the selection of the most suitable treatments for PALF across various applications, ranging from textiles to innovative materials.

### 3.4 Physical-mechanical properties of Azores PALF

Understanding physical-mechanical properties of PALF is crucial for their effective application in advanced textile materials. Extracted PALF can vary in properties regarding the variety of the pineapple, region of cultivation, age of the plant, extraction method, among other factors. These variations typically include a diameter range of 5–280 μm [42,71], tensile strength ranging from 170 to 1627 MPa, Young's Modulus ranging from 4.2 to 84.5 MPa, and elongation at break ranging from 1.4 to 4.0% [24]. As of the present date, there appears to be a lack of published data on the physical-mechanical properties of PALF from the Azores Island.

In general, a significant variability in the physical-mechanical properties of Azores PALF was observed. A wide range of values is evident, as shown in Table 2, Table 3. Such variability is an intrinsic characteristic of natural fibers, and consequently for PALF [74]. Neto et al. observed differences in PALF properties among six varieties of the same pineapple species (*Ananas genus*) [33]. Da Paixão et al. demonstrated that Indian PALF from the same species and regions (*Ananas comosus*) varies in mechanical properties, with a 59% variation in tensile strength [75]. Surajarusarn et al. reported significant standard deviations for tensile properties (i.e., tensile strength, Young's modulus, and elongation at break) and cross-section of PALF from different parts of the pineapple leaf [34]. The inherent variability in PALF properties may present challenges in achieving uniformity in product properties and quality. Overcoming this challenge requires optimizing processing techniques to minimize variations in fiber properties. Exploring methods such as more sustainable chemical treatments or mechanical processing can enhance overall uniformity in fiber characteristics [76]. Effective homogenization through carding machines and blending techniques, which involve combining fibers from diverse sources, can contribute to the uniformity, reliability, and performance of end-products in the textile industry, including yarns and non-woven textiles [17,57].

**Table 2:** Effect of the extraction method, plant age and different regions of the bundles over the PALF diameter.

Fibers samples	Region of the individual fiber	Range of diameters (μm)
Hand scraped (18 months)	Beginning	54.0–121.3 (80.3)^a^
	Middle	52.7–133.4 (86.8)
	Tip	35.1–111.7 (66.0)
Hand scraped (11 months)	Beginning	36.1–147.4 (86.2)
	Middle	55.3–155.6 (94.8)
	Tip	40.5–118.2 (68.9)
Retted at 25 °C (7 days)	Middle	43.5–171.2 (87.3)
Retted at 25 °C (14 days)	Middle	34.9–168.3 (79.1)
Retted at 25 °C (21 days)	Middle	34.3–144.4 (77.3)
Retted at 25 °C (28 days)	Middle	37.3–111.3 (66.6)
Literature	–	5,0–280,0^b^

a Values between parenthesis represent the average of the range.

b [42,71].

**Table 3:** Physical-Mechanical properties of PALF with different ages extracted by hand scraping.

Fibers samples	Fibers Length (cm)^1^	Linear Density (tex)	Position in the leaf	Tensile strength (MPa)	Elongation at break (%)	Young's modulus (GPa)^2^
Hand scraped (18 months)	19.6–52.5	1.68–6.59	Beginning	43–322	0.9–3.4	4.8–16.2
			Tip	44–295	0.9–3.1	4.4–20.4
Hand scraped (11 months)	26.9–49.0	1.49–6.04	Beginning	56–529	0.9–4.2	4.3–16.9
			Tip	32–439	0.5–3.2	2.4–21.3
[24]	–	–	–	127–1627	1.4–4.0	4.2–84.5

^1^Corresponds to the specimens randomly separated for physical-mechanical testing. ^2^R^2^ was >0,99 for all the specimens.

#### 3.4.1 PALF diameter

**Fig. 8:**
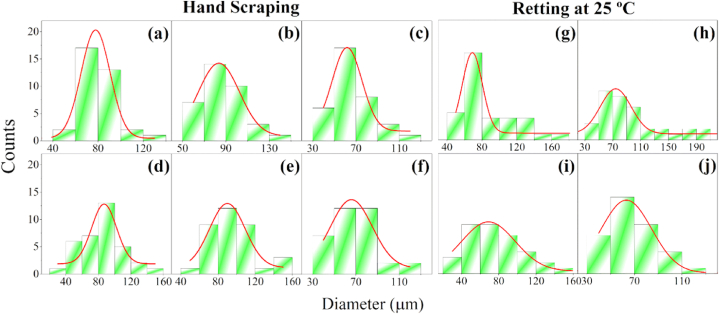
Histograms of diameters distribution of the extracted fibers.

The histograms of the diameters distribution of the extracted fibers are observed in Fig. 8. It can be observed that PALF vary in a wide range of dimeters, which is expected for natural fibers [77].

Fiber diameter plays an important role in textile applications. The effects of the studied extraction methods, plant age, and fiber region on the diameter of PALF are presented in Table 2. In general, the diameter of untreated PALF from Azores pineapple plants was in the range of 34.3–171.2 μm, which agrees with those already reported in literature (*i.e*., 5–280 μm) for different varieties, mostly falling within the lower half of this range [24,42,71].

A noticeable pattern emerges when comparing the average diameter of fibers extracted from different positions in the leaf, with fibers showing a slightly increase in average diameters from the beginning to the middle, and then decreasing toward the tip. Similar findings were reported by Surajarusarn et al. who observed that the average cross-sectional area of hand-scraped PALF bundles, from Thailand pineapple plants, gradually decreases from the base to the tip of the leaf [34]. Kaewpirom and Worrarat also observed this same pattern for mechanically extracted PALF [49] and it was attributed to the different components chemical composition [78]. Variations in the morphology, such as diameter, and mechanical properties of natural fibers can be correlated with differences in the chemical composition of fibers, attributed to maturation time. For example, during the maturation of the kenaf plant, there is a significant increase in cellulose and lignin content [79]. The cellulose content of woody plants also increases with age [59]. As indicated by the results of the DTG analysis (see Fig. 4-a), a lower cellulose content was observed for 11-age PALF extracted from the tip of the leaf. This is likely due to the shorter maturation time of fibers in this particular position of the leaf, which makes the fiber thinner at this position. This trend in fiber diameter is consistent across different plant ages (*i.e*., 11 and 18 months). However, differences in fiber diameter among the studied ages were remarkable.

For PALF extracted through the retting procedure at 25 °C, both the average and maximum diameter decreased over the time (Table 2). This reduction is attributed to the enzymatic and bacterial removing of gum, hemicellulose, lignin, cellulose, and other components in the fiber, as reported by Ref. [80]. While retting is employed for fiber extraction reducing the diameter in an environmentally friendly way, prolonged retting may negatively impact fiber quality. This was confirmed through ATR-FTIR and TG/DTG analysis, which demonstrated undesirable effects after 21 days of retting, ultimately affecting the tensile properties of PALF, as discussed further below.

#### 3.4.2 Tensile properties of Azores PALF

The physical-mechanical properties of PALF extracted by the hand scraping method are exhibited in Table 3**.**

The tensile behaviour of natural fibers is correlated to their morphology [81]. Since we noticed a high similarity in morphology between fibers from the middle and beginning positions in the leaf, a comparison was made of the tensile properties between the beginning and tip positions of the leaf only. As demonstrated by Surajarusarn et al. (2019) through their Weibull plot analysis, fiber from these both positions have a similar distribution for tensile-strength properties [34]. The tested specimens had lengths of up to 52.5 cm and showed linear density in the range of 1.49 and 6.59 tex. Mechanical properties exhibited a large range, as expected for natural fibers [23], with tensile strength ranging from 32 to 529 MPa, elongation at break from 0.5 to 4.2 GPa, and Young's modulus from 2.4 to 21.3 GPa. These values are consistent with those previously reported in the literature for different varieties of pineapple [24], standing in the first half of the range.

It is evident that higher tensile strength values were found for fibers extracted from the initial part of the leaf. These findings corresponded to a higher diameter of the fibers from this same position in the leaf, regardless of the age of the plant (i.e., 11 and 18-months). Similar results were found by Surajarusarn et al. (2019) for tensile strengths and cross-section areas of fibers extracted from different parts of pineapple leaf from cultivations in west Thailand.

Nevertheless, the differences between ages are not remarkable. The specimens from plants with 11 months of age reached higher tensile strength values. However, although no shown here, the average values of these ranges (*i.e*., 11 months: 190 and 155 MPa, 18 months: 203 and 181 MPa, beginning and tip, respectively) were also of little significance. These findings reveal an advantage in using pineapple waste for cellulosic fiber extraction. Since the age of the plant within a range of 7 months has little consequence on PALF mechanical properties, it is possible to take advantage of more heterogeneous waste fibers or even those generated before pineapple harvest, such as pruning waste, for instance.

The physical-mechanical properties of PALF extracted by the retting method at 25 °C are exhibited in Table 4, for the fibers which shown the bigger and the smaller cellulose DTG peak in DTG analysis (*i.e*., 14 and 28-days in retting process).

**Table 4:** Physical-Mechanical properties of PALF extracted before and after 14 and 28 days of Retting at 25 °C.

Fibers samples	Fibers Length (cm)^1^	Linear Density (tex)	Tensile strength (MPa)	Elongation at break (%)	Young's modulus (GPa)^2^
0 day	26.9–49.0	1.49–6.04	64–397	1.2–3.3	3.6–13.1
14 days	29.3–70.8	1.39–7.07	37–993	1.0–3.9	2.4–21.8
28 days	23.9–44.2	1.26–6.52	53–395	0.8–3.3	4.6–23.0

^1^Corresponds to the fibers separated for physical-mechanical testing, including the bigger one in the bundle, not the smaller. ^2^R^2^ was >0,99 for all the specimens.

The length of the tested specimens was up to 70.8 cm, with a linear density ranging between 1.26 and 7.07 tex. The tensile strength of PALF from retted leaves ranged from 53 to 516 MPa, elongation at break from 0.9 to 3.4 GPa, and Young's modulus from 3.6 to 23. Following the same pattern observed for cellulose DTG peak and ATR-FTIR absorption bands intensity, tensile strength improved until 14 days of retting and decreased after 28 days of retting (see maximum values in the range, Table 4). This indicates efficient removal of non-fibrous matter during the 14-day retting process, but longer periods of retting led to damage to the cellulosic fiber due to cellulose decomposition. Notably, from 14 days of retting, it was possible to extract the longer fibers (i.e., 70.8 cm), as the removal of non-fibrous matter and improved cellulose content prevented fibers from breaking during the scrapping process. Since some leaves have reached a length of 96.5 cm, they can accommodate fibers as long as 70.8 cm.

**Fig. 9:**
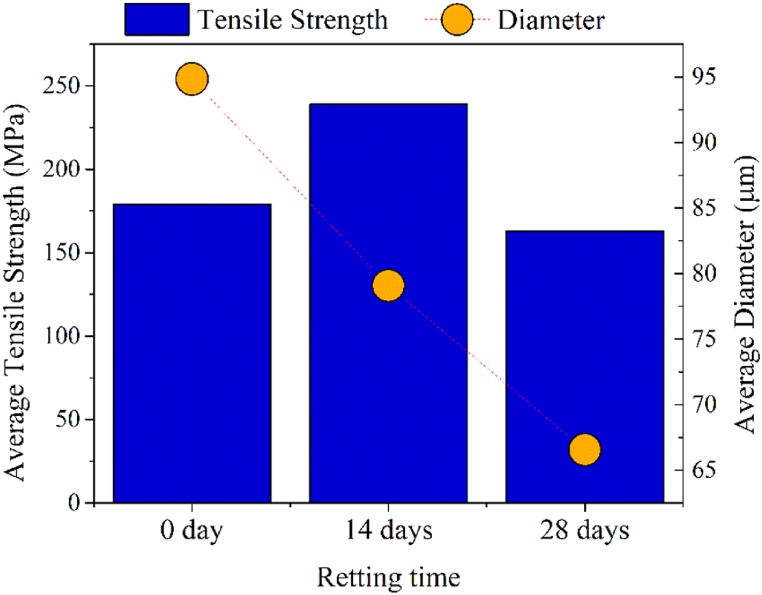
Relationship between tensile strength and diameter of Azores PALF extracted by retting at 25 °C.

Tensile strength has an intimate relationship with fiber diameter in natural fibers [82]. The effect of the retting time at 25 °C on the relationship between fiber diameter and its tensile strength can be clarified through Fig. 9.

While increasing retting time has gradually reduced the fiber diameter, tensile strength improved up to 14 days of retting when the removal of non-fibrous matter reached its maximum without damaging the fibers. The removing of non-cellulosic materials not only reduces the fiber diameter, but also exposes more cellulose hydroxyl groups. These exposed hydroxyl groups can participate in additional hydrogen bond formation, strengthening the interactions between cellulose chains and fiber strength [68].

This result corroborates the procedure adopted by other researchers in the extraction of PALF from Indian varieties of pineapple. Jain and Sinha extracted PALF for chemical treatments to reinforce polymer composites, using the same procedure of soaking leaves in water for 15 days [83]. Jagadish et al. extracted PALF to make composites based on resins by soaking pineapple leaves in water for two weeks [84]. These authors also highlighted that long periods of retting can damage the fiber and lower its strength.

## 4 Conclusions

Long fibers, measuring up to 70.6 cm in length, were successfully extracted from Azores pineapple leaves through eco-friendly methods, including hand scraping and biological retting at 25 °C, across 11 and 18-months plant ages. The linear density of Azores PALF ranged from 1.26 to 7.07 g km^−1^, with tensile strength varying from 37 to 993 MPa, elongation at break ranging from 0.5 to 4.2 %, and Young's modulus from 2.4 to 23 GPa.

These fibers exhibited distinct properties along different regions, being thicker and stronger in the initial segment, regardless of the plant age. Due to their length, specific regions of these fibers can be selected for more suitable applications through chopping. Moreover, the difference between the 11- and 18-months age groups was negligible, indicating that the waste material can be utilized not only after fruit harvesting but also after pruning.

Among the studied extraction methods, the 14-day biological retting process at 25 °C was able to remove non-fibrous matter yielding fibers with superior mechanical characteristics (i.e., 29.3–70.8 cm longer, 37–993 MPa tensile strength, 1.0–3.9 % elongation at break and 2.4–21.8 MPa Young's Modulus). XRD analysis revealed no differences in the crystallinity index of cellulose fraction suggesting preservation of hemicellulose and lignin, which can be changed with further pre-treatments depending on the intended application of Azores PALF.

These findings demonstrate that Azores pineapple waste represents an immensely advantageous resource to address the lack of a PALF supply chain in Portugal and Europe, providing high-quality fibers for the textile industry and contributing to the improvement of the local circular economy.

## CRediT authorship contribution statement

**Diego M. Chaves:** Writing – original draft, Visualization, Methodology, Investigation, Conceptualization. **Joana C. Araújo:** Writing – review & editing, Visualization, Validation. **Carina V. Gomes:** Methodology, Investigation. **Sónia P. Gonçalves:** Methodology, Investigation. **Raul Fangueiro:** Supervision, Project administration, Funding acquisition. **Diana P. Ferreira:** Writing – review & editing, Visualization, Supervision, Project administration, Funding acquisition, Conceptualization.

## Declaration of competing interest

The authors declare that they have no known competing financial interests or personal relationships that could have appeared to influence the work reported in this paper.
